# Herpesviruses and Intermediate Filaments: Close Encounters with the Third Type

**DOI:** 10.3390/v3071015

**Published:** 2011-07-04

**Authors:** Laura Hertel

**Affiliations:** Center for Immunobiology and Vaccine Development, Children’s Hospital Oakland Research Institute, 5700 Martin Luther King Jr. Way, Oakland, CA 94609, USA; E-Mail: lhertel@chori.org; Tel.: +1-510-450-7989; Fax: +1-510-450-7910

**Keywords:** intermediate filament, herpesvirus, herpes simplex virus, varicella zoster virus, cytomegalovirus, Epstein-Barr virus, Kaposi’s sarcoma associated herpesvirus, cytoskeleton, infection

## Abstract

Intermediate filaments (IF) are essential to maintain cellular and nuclear integrity and shape, to manage organelle distribution and motility, to control the trafficking and pH of intracellular vesicles, to prevent stress-induced cell death, and to support the correct distribution of specific proteins. Because of this, IF are likely to be targeted by a variety of pathogens, and may act in favor or against infection progress. As many IF functions remain to be identified, however, little is currently known about these interactions. Herpesviruses can infect a wide variety of cell types, and are thus bound to encounter the different types of IF expressed in each tissue. The analysis of these interrelationships can yield precious insights into how IF proteins work, and into how viruses have evolved to exploit these functions. These interactions, either known or potential, will be the focus of this review.

## 1. Introduction

Intermediate filaments (IF) constitute the third main cytoskeletal system of vertebrate cells. They not only provide structural and mechanical support to the cell, but are also involved in multiple cellular functions, including transport, protein and organelle targeting, migration, signaling, apoptosis, and protection from stress [1–4]. Because of this, IF are likely to play essential roles in enabling or restricting pathogen infection, and have the potential to become useful targets for new antiviral therapies. The dissection of IF-virus interactions could also provide crucial insights into the molecular mechanisms underlying the more than 80 human IF-linked diseases, and into the workings of cellular processes with the potential to be exploited therapeutically, such as the intracellular trafficking and delivery of cargoes. Compared to our knowledge of how viruses utilize the other two cytoskeletal components, the microfilaments (MF) and microtubules (MT), however, our current understanding of IF-virus interactions is largely incomplete. This review will focus on the interrelationships between human herpesviruses and IF during virion entry and egress, and during viral genome replication.

### 1.1. IF: An Overview

With an average diameter of 10 nm, IF owe their name to being of “intermediate” size between MF (6 nm in diameter) and MT (25 nm in diameter). Numerous types of IF are formed by the assembly of different polypeptides, displaying common structural domains but distinct expression profiles (Table 1). Because of their tissue-specific functions, the 70 currently known human IF proteins have been associated with a number of pathologies affecting various organs including the skin (e.g., epidermolysis bullosa simplex), the heart (e.g., dilated cardiomyopathy), the cornea (e.g., cataract), muscles (e.g., muscular dystrophy) and neurons (e.g., Charcot-Marie-Tooth disease) [4,5].

IF monomers consist of an alpha-helical rod flanked, at each end, by globular domains. Monomer dimerization is followed by the formation of tetramers via dimer association in antiparallel orientation, a setting that gives rise to smooth filaments devoid of polarity [6–8]. The polymerization of soluble, nucleotide-bound actin or tubulin, by contrast, generates polarized filaments possessing a plus end, to which subunits are added, and a minus end, from which subunits can be removed in a process known as treadmilling [9,10]. In contrast to MF and MT, IF assembly: (i) occurs spontaneously and in the absence of ATP or GTP [6–8,11]; (ii) does not depend on nucleotide availability or on the presence of cofactors and regulators, albeit new proteins facilitating IF polymerization have recently been identified [12]; (iii) is primarily, although not exclusively, regulated by phosphorylation [13]; and (iv) cannot be blocked by any currently available pharmaceutical compound.

IF are durable, stable and remarkably more resistant to breakage than MF and MT [14]. Despite this, their intracellular networks are far from being static, and can quickly reorganize in response to a variety of extra- or intracellular stimuli. The molecular mechanisms at the basis of IF dynamics appear to depend on the kind of IF, the type of cell, and the specific intracellular location of filaments [8]. Lamin, vimentin and neurofilament polymerization, for instance, occurs at a slower rate than that of keratin [15], with the majority of nascent filaments moving in anterograde direction toward the cell surface. Keratin assembly, on the contrary, occurs principally at the cell periphery, and newly formed filaments are continually shifted toward the cell center [16]. The presence of intact MF and MT is required to support these movements, as transport of IF precursors relies on motor proteins associated with MT (kinesins and dyneins), and MF (myosins) [17–22]. All three cytoskeletal systems are thus largely interconnected, both functionally and physically. Vimentin IF and MT are tightly linked in human fibroblasts [23], with the pharmacological fragmentation of MT leading to the collapse of vimentin networks [17,23–25]. Bundling of vimentin IF by antibody injection, however, did not alter MT structure, indicating that MT-IF interactions are functionally unidirectional [26–28].

Contrary to MF and MT, IF lack polarity and are not exploited as rails for intracellular transport. IF do, however, participate in a variety of trafficking events, including sorting of endo-lysosomes, mitochondria, and Golgi stacks [29], targeting of proteins to specific locations [29], and transmission of signals from the periphery to the nucleus for gene expression control [30]. IF are also crucial for proper shaping [31], positioning [32] and anchoring [33] of the nucleus, and to reduce the impact of mechanical and other types of stress on key cellular activities [13].

### 1.2. Human Herpesviruses and Their Tropisms

Herpes simplex virus 1 and 2 (HSV-1 and HSV-2), varicella zoster virus (VZV), human cytomegalovirus (CMV), human herpesvirus-6 and -7 (HHV-6 and HHV-7), Epstein-Barr virus (EBV) and Kaposi’s sarcoma associated herpesvirus (KSHV) comprise the eight currently known human members of the *Herpesviridae* family (Table 2). All share a similar virion structure, consisting of a linear, double-stranded DNA molecule densely packaged into an icosahedral capsid, with a diameter of 115–130 nm. The capsid is surrounded by an amorphous protein layer, called the tegument, consisting of more than 30 proteins of viral and cellular origin, and by a lipid envelope containing approximately 10–12 viral glycoproteins plus a few cellular polypeptides [34]. Infectious virions are spherical, and measure approximately 200 nm in diameter.

All herpesviruses are species-specific, but the range of cell types and tissues they can infect within each species varies widely. *In vitro*, HSV-1 and HSV-2 can enter most primary and established cells lines, while *in vivo* their tropism is highly restricted to the epithelial cells of the oropharyngeal, respiratory and genital mucosae and to the innervating sensory neurons, where they establish latency [35,36]. In contrast to HSV, the range of cells permissive to VZV infection *in vitro* is extremely limited, whereas *in vivo* this virus can infect not only epithelial cells and neurons, but also monocytes, dendritic cells, and T and B lymphocytes [37].

Almost all cell types, with the notable exception of lymphocytes and polimorphonuclear leukocytes, are permissive to CMV infection *in vivo* [38]. Consequently, virtually all organs have been found to harbor replicating virus in acutely infected individuals [39,40]. Among permissive cell types, fibroblasts, epithelial, endothelial, smooth muscle and dendritic cells are particularly relevant to the development of disease, acting as sites of virus amplification, spread and immune evasion [38,39,41–45], while CD34^+^ hematopoietic progenitors and monocytes are major reservoirs of latent virus [38,46]. *In vitro* HHV-6 replicates most efficiently in activated CD4^+^ lymphocytes, but can infect additional cell types including NK, liver, epithelial, endothelial and dendritic cells, fibroblasts, fetal astrocytes, and microglia [47]. *In vivo*, this virus is frequently detected in CD4^+^ T cells and endothelial cells, brain and liver tissue, tonsils and salivary glands and, in latent form, in monocytes [47–49].

Although B lymphocytes and epithelial cells are the main targets of EBV infection, numerous other cell types can be infected *in vivo*, including T lymphocytes, dendritic cells, NK and smooth muscle cells. KSHV’s tropism appears to be broader than that of EBV, both *in vitro* and *in vivo*, with B cells, endothelial cells, epithelial cells, keratinocytes and fibroblasts all capable of supporting production of viral progeny [50]. Both viruses establish lifelong latency in resting memory B cells [51].

Owing to their broad tissue tropism, herpesviruses are bound to encounter virtually all types of IF during infection *in vivo*. Despite this, our current knowledge of these interactions is still largely limited to just a few IF proteins.

## 2. Intermediate Filaments in Herpesvirus Entry

**Figure 1:**
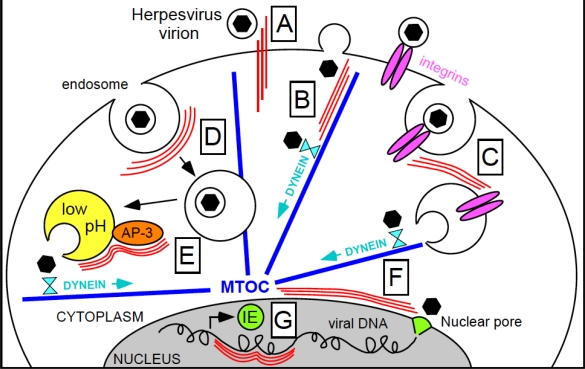
Steps during herpesvirus entry that may require intermediate filaments’ assistance for efficient completion. IF and microtubules are shown as three parallel thin red lines and as thick blue lines, respectively. Black hexagons enclosed in a circle depict enveloped virions, while isolated black hexagons represent virus capsids. (**A**) Keratin-type IF potentially strengthening initial virion interactions with the cell surface; (**B**) enhancement of capsid attachment to microtubules via IF; (**C**) internalization of integrin-bound virions under the control of IF; (**D**) endosomes trafficking towards the cell center accompanied by IF; (**E**) AP-3 mediated endosome acidification and viral particles release facilitated by IF; (**F**) capsid movement along the nuclear envelope towards the nuclear pore complex assisted by IF; and (**G**) regulation of viral gene transcription onset by nuclear IF. MTOC, microtubule organizing center.

Herpesviruses replicate their genomes in the nucleus. To start productive infection, therefore, penetrated virions must be actively transported from the cell periphery to the nuclear compartment. This is accomplished in essentially two ways, depending on the virus mode of entry [52]. If penetration occurs by fusion of the viral envelope with the plasmalemma, “naked” virions, *i.e.*, capsids and tightly associated tegument proteins, are transported toward the nuclear envelope along cellular MT [53–55]. If, by contrast, viral entry occurs by endocytosis or macropinocytosis, the initial movement of virions toward the cell center takes place within cellular vesicles. Upon fusion of the viral envelope with vesicular membranes, capsids are then released into the cytosol, and reach the perinuclear space by associating, once again, with MT [36,51,52]. The long journey virions endure to enter host cells and reach the nucleus provides IF with numerous opportunities to support or hinder infection initiation (Figure 1).

### 2.1. Virion Binding at the Cell Surface

Natural transmission of herpesviruses occurs by contact between virions present in the bodily fluids of infected individuals and the oral or ano-genital mucosae of a new host. The epithelial cells and keratinocytes lining these openings express different types of keratins, while fibroblasts and endothelial cells in the underlying connective tissues contain vimentin. Both proteins can be exposed at the cell surface, where they may participate in cell-pathogen interactions.

Keratins are deposited outside of the cell in specific areas of the mouth such as the hard palate, the gingiva, the tongue and the outer lip [56]. Their presence at these sites usually provides an effective barrier against pathogen invasion, although several bacteria were shown to use surface keratins as host-binding anchors [57–61]. Although the actual presence of non-keratin types of IF at the cell surface is still under debate and a matter of controversy, extracellular exposure of vimentin was reported to favor attachment and internalization of viruses such as the porcine reproductive and respiratory syndrome virus [62], human immunodeficiency virus-1 [63], and Theiler’s murine encephalomyelitis virus [64]. As the entry process of most herpesviruses, except EBV, begins with virion attachment to the ubiquitous heparan sulfate proteoglycans [65], surface keratins are unlikely to act as primary sites of virion binding to the cell surface, although a role for these proteins in strengthening these initial virus-host contacts remains possible (Figure 1A). Primary HSV infection of the oral cavity generally occurs within non-keratinized tissues [66], and in an organotypic culture system mimicking stratified human epithelia, successful initiation of HSV-1 infection required virions to slip through microabrasions in the keratinized suprabasal layers of the epithelium [67]. At least for HSV, thus, keratins appear to hinder, rather than promote, virion entry. Whether surface vimentin plays any role in supporting initiation of herpesvirus infection, by contrast, remains to be determined.

### 2.2. Virion Penetration

All herpesviruses enter host cells by fusion of the viral envelope with cell surface or vesicular membranes, depending on the complement of proteins present on the virion and on the cell type [52]. CMV entry into epithelial and endothelial cells, for instance, occurs by macropinocytosis followed by capsids release in a low-pH-dependent [68,69] or independent [70,71] way, and requires the presence of the gH/gL/UL128/UL130/UL131A complex on the viral envelope [43,72,73]. CMV strains lacking this complex have a more limited tropism, and enter cells by fusion at the plasma membrane [38]. Engagement of cellular vesicles is also required for entry of HSV into epithelial cells such as keratinocytes and HeLa cells, and of EBV into B cells, while penetration of HSV into fibroblasts, neurons and African green monkey kidney epithelial cells, and of EBV into primary human foreskin epithelial cells occurs by fusion at the cell surface [74–77].

All herpesviruses enter host cells by fusion of the viral envelope with cell surface or vesicular membranes. Irrespective of the mechanism used, herpesvirus entry usually leads to MF remodeling, accompanied or not by filament disassembly, while the structural integrity of MT is generally maintained to support capsid transport toward the nucleus [78–87]. Currently available data also point at a remarkable conservation of IF networks [83–85,88,89], suggesting that the integrity of this system may be required for viral entry. In a recent work, we tested this hypothesis using human fibroblasts and two different CMV strains, one entering cells by fusion at the plasma membrane (AD169) [90] and one expected to enter by fusion with both surface and vesicular membranes (TB40/E) [83]. Penetration of both types of virions was severely impaired in cells where the vimentin cytoskeleton had been disrupted with acrylamide, a neurotoxin causing IF collapse in perinuclear aggregates [25]. Onset of infection was also delayed (AD169), and both delayed and reduced (TB40/E), by vimentin bundling in fibroblasts from patients with giant axonal neuropathy. Finally, the complete absence of this protein in vimentin^−/−^ mouse embryo fibroblasts deferred AD169 particle translocation across the cytosol, and completely blocked progression of TB40/E virions [83]. These results show that the presence of an intact vimentin network is required for CMV infection onset, and hint at a differential role for these IF in supporting virion progress toward the nucleus. Virion entry by fusion at the cell surface, indeed, appeared to be less dramatically affected by the absence of vimentin than entry by macropinocytosis, suggesting that vimentin’s role in managing intracellular vesicles’ movement may be more relevant to CMV infection than its scaffolding functions at the cell periphery.

Whether other types of IF are required for viral entry in clinically-relevant cells like epithelial cells and neurons, and whether the presence of intact vimentin networks is also necessary for infection with other herpesviruses besides CMV remains to be determined. Also to be explored is the precise role played by IF during herpesvirus entry, a task complicated by the fact that the exact function of numerous IF proteins has not yet been established. Based on current knowledge, however, the following possibilities may be envisaged: (i) being tightly connected with MT, some IF proteins might facilitate binding of penetrated capsids to MT motor proteins at the cell periphery (Figure 1B); (ii) vimentin interacts with integrins α2β1, α6β4 and αVβ3 at the cell surface, and mediates recycling of integrin-containing endocytic vesicles [91,92]. These IF could thus support entry of herpesviruses that use integrins as receptors, such as CMV [93–95], KSHV [96,97], and EBV [98], by enhancing the internalization of vesicles containing virion-integrin complexes (Figure 1C); (iii) vimentin also accompanies endocytic vesicles in their migration toward the nucleus [99] and, together with the glial fibrillary acidic protein (GFAP), promotes the directional mobility of vesicles in astrocytes [100]. These functions could thus be exploited by endocytosed viral particles to traffic from the periphery to the center of the cell (Figure 1D). Vimentin’s role in stimulating clathrin uncoating from endocytic vesicles [101], by contrast, may not have an impact on infection onset, as herpesvirus virions are considered to be too large to fit into clathrin-coated endosomes, with the notable exception of KSHV [87,102]; (iv) finally, vimentin, peripherin and α-internexin also bind to AP-3 [103], an adaptor complex that regulates trafficking of vesicles in the endo-lysosomal compartment [104], maintains endo-lysosomal stores of cellular ionic zinc in fibroblasts [105], and controls the intracellular localization of the chloride channel ClC-3 [106], thus potentially contributing to influence lysosomal pH [107,108]. In the absence of vimentin, lysosomes are small, do not properly acidify, and redistribute from their usual disperse cytoplasmic location to a juxtanuclear region [101,103]. Thus, at least in those instances where release of internalized capsids from cellular vesicles is pH-dependent [68,69,74,102,109,110], the presence of vimentin, peripherin or α-internexin may be required to induce the rapid acidification of endo-lysosomes via AP-3 (Figure 1E). Both vimentin and keratins, however, also appear to have a role in stimulating the formation of autophagosomes, as disruption of hepatic keratin networks completely inhibited autophagy [111], while vimentin^−/−^ cells were shown to contain lower number of autophagocytic vesicles than wild-type cells [103]. This specific IF function may thus favor the host, rather than the virus, by fostering the active degradation of penetrated particles. Herpesviruses have indeed evolved a number of strategies to manipulate this cellular defense mechanism, although none have yet been reported to affect IF’s participation in this process [112].

### 2.3. Viral Genome Deposition into the Nucleus

Dynein-bound capsids traveling along MT [113] are thought to reach the MT-organizing center (MTOC), where most MT plus ends are anchored [114]. This organelle is usually, but not always, located in proximity of the nucleus, at distances spanning from ∼1.5 μm in fibroblasts [115] to 5–10 μm in neurons [116]. Exactly how capsids reach the nuclear envelope across this gap, corresponding to about 10- to 80-fold their average size, remains unclear. Although most capsids are initially located close to the MTOC, their distribution becomes more evenly dispersed around the nucleus at later times [117]. The precise mechanisms mediating this spread are also currently unknown.

Electron microscopy imaging of HSV-1 and of murine gammaherpesvirus 68 entry into mammalian cells revealed that, once within 40 nm from the nuclear pore complex (NPC), capsids attach to the filaments radiating from the pore’s outer rim [117,118]. This binding step is followed by the translocation of the viral genome from the capsid into the nucleus through the capsid portal and the NPC, in a process that requires energy and the presence of cellular proteins other than those comprising the nuclear pore, and whose identity is currently unknown [119–121].

Cytoplasmic IF form cage-like webs around the nucleus, and can tightly associate with the nuclear envelope and with nuclear IF through a series of mediator proteins, including the recently discovered SUN (Sad1 and UNC-84) and KASH (Klarsicht, Anc-1 and Syne/Nesprin Homology) family members [122–128]. Thus, nuclear lamins bind to SUN protein dimers located in the inner nuclear membrane (INM). These, in turn, interact with KASH domain-containing proteins residing in the outer nuclear membrane (ONM). One of these proteins, nesprin-3, then completes the link between the nucleus and the IF cytoskeleton by interacting with plectin, an IF-binding protein [129,130]. At least in the case of vimentin, this tight grip of IF on the nuclear envelope was shown to be essential for maintenance of a rounded nuclear shape [31], for anchoring of the nucleus within the cell [131], for force transfer from the periphery toward the cell center [132], and to prevent nuclear spinning [33]. By forming a crate of filaments surrounding the nuclear envelope, IF are likely to dock to the nuclear membrane at multiple locations. Thus, IF could potentially support not only the transfer of capsids from the MTOC to the nucleus, but also their spread along the nuclear envelope, in order to reach NPCs located at greater distances from the centrosome (Figure 1F). Evidence in favor of a role for vimentin in fostering uptake of HIV-1 preintegration complexes into the nucleus has indeed been provided [63].

## 3. Intermediate Filaments in Herpesvirus Replication

Following genome deposition into the nucleus, transcription of the viral immediate-early (IE) genes must occur to start productive viral replication, while their silencing is required to establish latency.

Several lamina proteins were recently shown to participate in gene expression control by recruiting transcription factors and chromatin modifying enzymes to the nuclear periphery, thus leading to the formation of a transcriptionally repressive environment that is interrupted only in proximity of the nuclear pores [133]. For instance, while lamin A itself can inhibit gene expression when artificially tethered to promoters, [134], the lamina-associated protein 2β mediates transcriptional silencing by stimulating histone H4 deacetylation after binding to histone deacetylase 3 [135]. The lamin B receptor protein (LBR), by contrast, supports heterochromatin anchoring to the nuclear envelope by interacting with the heterochromatin protein 1 and with deacetylated histones H3/H4 [136].

Regularly spaced nucleosomes displaying the typical markers of heterochromatin are also usually associated with latent viral genomes maintained in episomal form within the nucleus of specific cells [137]. This transcriptionally silent status is reversed during reactivation bouts, possibly via reductions in the number of genome-associated histones, or via histone acetylation. Although a few viral and cellular proteins with roles in controlling these events have already been identified, a number of questions regarding the mechanisms and players involved in viral genome silencing during latency remain open [137]. Because of the prominent participation of the nuclear lamina in mediating transcriptional repression, nuclear IF are highly likely to be involved in viral latency establishment and/or maintenance, although this possibility has not yet been explored. By contrast, a role for lamin A in supporting the viral lytic replication cycle has been demonstrated [138]. In the absence of this lamin, nascent replication compartments were no longer retained within transcriptionally active areas of the nucleus, leading to increased heterochromatinization of the viral genome, strong inhibition of IE protein expression, and markedly reduced viral yields [138].

Other types of IF besides the lamins could also, potentially, be involved in herpesvirus replication events, on account of their involvement in intracellular signaling cascades [29]. Keratin mutation, GFAP overexpression and desmin downregulation were indeed shown to lead to changes in cellular gene transcription [29,139–141]. Theoretically, thus, these IF proteins could affect viral gene expression during both productive and latent life cycles (Figure 1G).

Progression of herpesvirus infection is also associated with dramatic changes in transcription levels of numerous cellular genes, including those encoding IF proteins (Table 3). Although the relevance of these changes for viral replication has not been fully established, they are likely to contribute to the pathological consequences of infection. The impact of VZV infection on IF gene expression, for instance, appears to be highly cell type-dependent, with the majority of changes observed in infected human skin implants in SCID mice (Table 3) [142]. In this tissue, transcription of numerous keratin genes was down-regulated, providing a possible molecular basis for the development of the typical skin blisters observed in patients with chickenpox. Moreover, the concomitant up-regulation of vimentin expression may indicate the onset of epithelial-to-mesenchymal transitions. In contrast, CMV infection of fibroblasts appeared to induce the appearance of an opposite phenotype, with reductions in vimentin, syncoilin and keratin 19 expression, and induction of keratin 5, 13, 18, 85 and 86 synthesis [143]. CMV infection in human neural progenitor cells also leads to the marked down-regulation of GFAP expression, and to the skewing of these cells’ differentiation toward a nonneuronal lineage. Both events are likely to lead to the severe nervous system malformations observed in newborns with cytomegalic inclusion disease [144]. Finally, transcription of different keratin genes is also altered in tissues infected with EBV or KSHV [145–147], while expression of EBV LMP or EBNA4 proteins stimulated expression of vimentin [148–150], but the significance of these changes for viral pathogenesis is still unclear.

## 4. Intermediate Filaments in Herpesvirus Egress

After being filled with newly replicated viral genomes, nucleocapsids leave the nucleus by sequentially budding through the INM and ONM in the so-called envelopment-deenvelopment-reenvelopment process, followed by the acquisition of tegument proteins and envelope membranes from the trans-Golgi network and the ER in the cytoplasm. Mature virions are then incorporated into exocytic vesicles for release at the cell surface [153–155]. Once again, this itinerary provides herpesviruses with numerous opportunities to interact with a variety of IF proteins.

### 4.1. Nucleocapsid Egress from the Nucleus

Because of their size (115–130 nm), herpesvirus nucleocapsids cannot exit the nucleus through the NPC, whose functional internal diameter measures 38 nm in size. All other passageways to the cytoplasm necessarily entail crossing of the nuclear envelope, whose INM, however, is supported and protected by a dense IF network composed of A- and B-type lamins, and of their partners [156]. As destabilization of the nuclear lamina is required for capsids to reach the INM, all herpesviruses have been reported to induce changes in the structure and/or distribution of lamina components at late times post-infection. Although the exact mechanisms mediating these rearrangements are not completely understood, they are thought to depend, at least in part, on lamin phosphorylation events, similar to those leading to the dissolution of the nuclear envelope during mitosis [154].

At late times post-infection with HSV-1, the usually uniform distribution of lamin A/C and B at the nuclear rim becomes irregular and discontinuous. While a proportion of the lamin B and LBR proteins is relocated to a perinuclear cytoplasmic region, possibly corresponding to the ER [157,158], changes occurring to the lamin A/C proteins appear to be more conformational than positional [159]. Lamin A/C and B amounts were also reduced in infected COS-1 cells [157], while no change in lamin A/C and lamin-associated protein 2 levels were detected in infected Vero cells [160]. This suggests that degradation of lamina components is cell type-specific, and is not necessarily required for its disruption. Both lamin B and the lamina-associated protein emerin are phosphorylated during infection [158,161], leading to the loss of connections between emerin and the nuclear membrane [162]. Lamin A/C is also phosphorylated on multiple sites by the viral kinases US3 and UL13, at least *in vitro* [163,164]. Whether these phosphorylation events are sufficient to induce fragmentation of the lamina to the extent required for virions to access the INM is unknown. Expression of HSV-2 UL13 alone, however, was shown to almost completely recapitulate the changes in lamin A/C and B distribution observed in infected cells [164].

**Figure 2:**
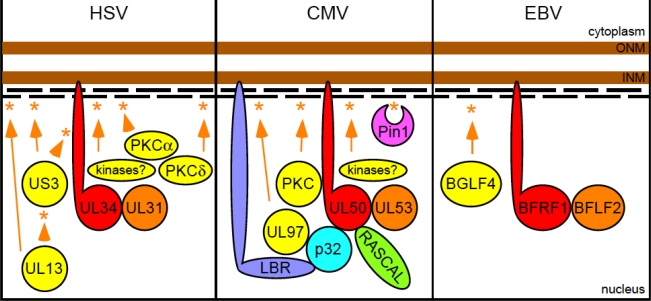
Herpes simplex virus 1 (HSV-1), cytomegalovirus (CMV) and Epstein-Barr virus (EBV) nuclear egress complex components and their interactions with the nuclear lamina. The two brown and thick lines represent the nuclear envelope, while the short black thin lines depict the lamin A/C and lamin B proteins comprising the nuclear lamina. Asterisks represent phosphorylation events performed by kinase proteins, whose names are contained in yellow-colored circles or ovals linked to the asterisks by orange arrows or arrowheads. The names of the two viral proteins composing the core of each nuclear egress complex (NEC) are contained within red- or orange-colored circles.

According to the current model, the HSV nuclear egress complex (NEC) consists of the following components [158–169] (Figure 2, left): a UL34-UL31 heterodimer, anchored to the INM via the transmembrane domain of UL34; the viral kinase UL13, which can phosphorylate US3, lamin A/C and lamin B, and regulates UL31 and UL34 recruitment to the INM; the viral kinase US3, which phosphorylates UL34 and lamin A/C, but is not necessary for lamina disruption, and may instead function as a negative regulator of the process; PKCα and PKCδ, which are recruited to the INM by the UL34-UL31 complex and are responsible for at least part of the observed phosphorylation of lamin A/C and B, and other, as yet unidentified, cellular kinases mediating emerin phosphorylation.

Quite interestingly, although lamins constitute a formidable barrier to nucleocapsid egress from the nucleus, and could thus be considered negative regulators of infection, HSV-1 titers are decreased (instead of increased) by at least 10-fold in *LmnB1*^−/−^ murine fibroblasts lacking expression of lamin B1 [170]. This suggests that lamin B is required for efficient viral replication, possibly because of its function in gene transcription and DNA replication [171,172]. Finally, although rearrangement of the nuclear lamina is required for efficient replication, it is possible for nucleocapsids to leave the nucleus in the absence of these changes, as mutant viruses lacking expression of UL31 and UL34 can still replicate to some extent in non-complementing cells [166]. Intriguingly, the nuclei of cells infected with bovine herpesvirus 1 were shown to contain large gaps through which nucleocapsids could reach the cytoplasm without budding into nuclear membranes [173]. Although the existence of these gaps is still a matter of debate, their generation may involve different kinds of interaction with the lamina than those mediating its dissolution, potentially requiring the activity of an alternative set of viral proteins.

CMV infection of human fibroblasts also profoundly modifies the structure of the lamina, inducing the appearance of deep INM invaginations that may favor capsid egress by acting as channels for the direct transport of virions towards the ONM [174–178]. The requirement for the presence of two viral proteins, M50 and M53, to achieve dissolution of the nuclear lamina was first demonstrated for murine CMV [179]. Their human CMV homologs, UL50 and UL53, were subsequently shown to be part of the CMV NEC (Figure 2, center), together with the viral kinase UL97, and the cellular proteins p32, LBR and PKC [180]. A new viral protein we just identified and named RASCAL also appears to localize to the NEC, likely through interactions with UL50 [82], while recruitment of the cellular peptidyl-prolyl *cis/trans* isomerase Pin1 to the nuclear rim was recently described to occur by binding to phosphorylated lamin A/C [181].

How, exactly, the nuclear lamina is disassembled during CMV infection is not completely understood. The complete disappearance of lamin A/C at late times post-infection has been described in some [182,183], but not other [82,184,185] reports. Whether proteolytic degradation of this lamin occurs and, if so, how much it contributes to the whole disassembly process is therefore still uncertain. Also unclear is whether expression of UL50 and UL53 alone can support these changes as described in COS7 cells [184], or whether their main function is to recruit other NEC components including the kinases PKC and UL97, whose activity, by contrast, is absolutely required for lamina disassembly [179–182,185–187]. Because of its role in lamin A/C phosphorylation, UL97 has been proposed to act as a viral analog of the cellular cyclin-dependent kinase 1 (CDK1) [188], which supports dissolution of the nuclear lamina during mitosis [189]. Interestingly, the appearance of pathological mitoses, characterized by the presence of multiple spindle poles and by the complete dissolution of the nuclear envelope, was observed to occur at late times post-infection with different strains of CMV, but in substantial amounts during infection with the fibroblast-adapted strain AD169 [190,191]. Pseudomitoses generation required the presence of CDK1, but the activity of this kinase was dispensable for the production and release of new viral particles [191]. By contrast, the simultaneous inhibition of multiple cyclin-dependent kinases, coupled or not to the inhibition of UL97, substantially reduced viral yields [191]. These results suggest that CMV may use a number of different cellular kinases, in addition to PKC and UL97, to support dissolution of the nuclear lamina during egress. Additionally, induction of pseudomitoses per se may represent an alternative method for CMV virions to leave the nucleus, since their presence in more than one quarter of infected cells did not have any negative effect on viral yields.

Finally, not much is known about the impact of EBV or KSHV infection on the nuclear lamina. In striking contrast to HSV and CMV infection, which cause a dissociation of the INM and ONM, EBV infection induces duplications of the nuclear membrane [192]. These were generated by the activity of the viral protein BFRF1, which localizes at the nuclear rim, interacts with lamin B and recruits a second viral protein, BFLF2, a putative modulator of BFRF1 activity [193–195]. Similar to the alpha and beta herpesviruses, however, EBV also appears to encode a nuclear rim-localized kinase, BGLF4, which can induce lamina disassembly by lamin A/C phosphorylation [154,196] (Figure 2, right).

### 4.2. Subviral Particle Trafficking from the Nucleus to the Cell Surface

After leaving the ONM, nucleocapsids must cross the cytoplasm to reach the cell surface. During infection with a variety of viruses, this step is associated with IF disassembly, possibly to enhance the speed of capsid movement by reducing the viscosity of the cytoplasm. Infection of epithelial cells with HSV-1 was indeed shown to cause partial proteolysis of keratins [197], while keratin 17 phosphorylation by HSV-2 US3 kinase was associated with reductions in these IF’s density [198]. However, considering the complexity of herpesvirus maturation in the cytoplasm, a role for IF in supporting, instead of hindering, herpesvirus egress is far more likely.

The interactions between vimentin, keratin and neurofilament IF and the Golgi apparatus [29], for instance, may be important for transfer of intracellular membranes to virions during their envelopment. In addition, desmin, keratins and neurofilaments are essential to maintain the correct morphology, distribution and function of mitochondria. In heart and skeletal muscles of desmin-null mice, mitochondria are misshapen and mislocalized, leading to a decrease in energy production and to the induction of apoptosis [199,200], while in neurons from patients with Charcot-Marie-Tooth disease caused by mutations in the neurofilament light subunit gene NF-L, mitochondria cluster close to the cell soma, leading to defects in axonal transport [201]. Through their interaction with mitochondria, thus, IF may support herpesvirus infection by securing an uninterrupted flow of energy, required to fuel viral genome synthesis and capsid transport. Also, IF may cooperate with viral anti-apoptotic proteins to prevent cell death. Finally, IF have a prominent role in targeting a variety of proteins to the plasma membrane [29], and in anchoring cells to each other as well as to the substratum by attaching to desmosomes and hemidesmosomes, respectively [202]. These functions could thus be exploited by maturing virions to promote the delivery of specific proteins to the sites of fusion between exocytic vesicles and the plasmalemma, and to target virion egress to the sites of cell-to-cell contact for lateral transmission within tissues. The NP protein of the lymphocytic choriomeningitis virus was indeed shown to promote intercellular spread of mature virions by binding to keratin 1 and stabilizing its filaments, leading to the establishment of stronger cell-to-cell contacts that favored viral transmission [203]. A similar role for keratins and other types of IF can be easily envisaged during herpesvirus egress, particularly so in the case of HSV and VZV, whose transmission and pathological manifestations involve keratinized organs such as the skin.

## 5. Concluding Remarks

In summary, IF are highly dynamic structures whose role in cellular physiology is just starting to be unraveled. The analysis of their functions during herpesvirus infections is certain to provide exciting new insights into how key steps of the viral cycle are completed, and is likely to lead to potentially new avenues in the treatment of herpesvirus infections.

## Figures and Tables

**Table 1:** Identity, tissue distribution and intracellular localization of intermediate filament (IF) family members.

**Type**	**Protein Name**	**Cell Type**	**Tissue Type**
I	Acidic keratins: K9–K28 K31–K40	Epithelial cells, keratinocytes	Mucosae, epidermis Hair, epidermal appendages
II	Basic keratins: K1–8, K71–80 K81–86	Epithelial cells, keratinocytes	Mucosae, epidermis Hair, epidermal appendages
III	Vimentin Peripherin Glial fibrillary acidic protein Syncoilin Desmin	Mesenchymal cells: fibroblasts, endothelial, hematopoietic cells Neuronal cells Astrocytes and glia Muscle cells	Connective tissue, blood, blood vessels Nervous system Nervous system Muscles
IV	α-internexin Neurofilament H, L, and M Nestin Synemin α, synemin β	Neuronal cells Neuronal cells Neuroepithelial cells Muscle cells	Nervous system Nervous system Nervous system Muscles
V	Lamin A, B1, B2, C1, C2	Ubiquitous	Ubiquitous
VI	CP49/phakinin, filensin/CP115	Eye lens cells	Eye lens

**Table 2:** Human herpesviruses and permissive cell types.

**Subfamily**	**Virus Name**	**Productive Infection**	**Latency**
Alpha	HSV-1	Epithelial cells, neurons	Neurons: trigeminal ganglia
	HSV-2		
	VZV	Epithelial cells, neurons, monocytes, dendritic cells, T and B lymphocytes	Neurons: dorsal root ganglia
Beta	CMV	Most cell types except lymphocytes, eosinophils, basophils, and neutrophils	Myeloid progenitors
	HHV-6	CD4+ T cells, neurons, astrocytes, microglia, fibroblasts, epithelial, endothelial and dendritic cells	Lymphocytes, Monocyte/macrophages, other?
	HHV-7	CD4+ T cells	
Gamma	EBV	B and T cells, dendritic, NK and smooth muscle cells	Memory B cells
	KSHV	B cells, endothelial cells, epithelial cells, keratinocytes and fibroblasts	

**Table 3:** Changes in expression levels of IF-encoding genes as reported in functional genomics analyses of infected human tissues and fibroblasts (HF).

**Gene Name**	**Gene Symbol**	VZV in T Cells *	VZV in Skin *	VZV in HF *	**HSV in HF ^&^**	**CMV in HF ^@^**

Keratin 1	KRT1	-	DOWN	-	-	-
Keratin 5	KRT5	-	DOWN	-	-	UP
Keratin 6A	KRT6A	-	DOWN	UP	-	-
Keratin 8	KRT8	-	-	-	UP	-
Keratin 13	KRT13	-	-	-	-	UP
Keratin 17	KRT17	-	DOWN	-	-	-
Keratin 18	KRT18	-	-	-	UP	UP
Keratin 19	KRT19	-	UP	-	-	DOWN
Keratin 33A	KRT33A	UP	-	-	-	-
Keratin 71	KRT71	-	DOWN	-	-	-
Keratin 85	KRT85	-	-	-	-	UP
Keratin 86	KRT86	-	-	-	-	UP

Desmin	DES	-	UP	-	-	-
Peripherin	PRPH	-	-	-	UP	-
Syncoilin	SYNC	UP	-	-	-	DOWN
Vimentin	VIM	-	UP	-	DOWN	DOWN

Neurofilament 3	NEF3	-	DOWN	-	-	UP
Neurofilament heavy	NEFH	DOWN	-	-	-	-

Lamin B1	LMNB1	-	-	-	-	UP

Data derived from the following references:

* [142];

& [151,152];

@ [143].
